# Editorial: Transcranial electrical stimulation (tACS, tDCS, tRNS) in basic and clinical neuroscience: current progress and future directions

**DOI:** 10.3389/fnhum.2025.1640565

**Published:** 2025-06-20

**Authors:** Desmond Agboada, Carmelo Mario Vicario, Miles Wischnewski

**Affiliations:** ^1^Department of Psychology, University of the Bundeswehr Munich, Neubiberg, Germany; ^2^Department of Psychology, University of Messina, Messina, Italy; ^3^Department of Psychology, University of Groningen, Groningen, Netherlands

**Keywords:** neuroplasticity, tACS, tDCS, tRNS, long-term effects, clinical trials

Transcranial electrical stimulation (tES) as a non-invasive brain stimulation technique has been used to study brain physiology for many years now (Nitsche and Paulus, 2000; Antal et al., 2017). Within this period, rapid advancement in understanding its mechanisms of action (Liu et al., 2018; Jackson et al., 2016; Yavari et al., 2018) and optimization of neuromodulatory effects have taken place (Agboada et al., 2019, 2020; Mosayebi Samani et al., 2019a,b; Wischnewski et al., 2019), with evidence from healthy and clinical populations (Alizadehgoradel et al., 2024; Ney et al., 2021; Vicario and Nitsche, 2013). The tES methods, including transcranial direct current, alternating current, and random noise stimulation (tDCS, tACS, and tRNS), operate via the application of weak currents through electrodes on the scalp with the aim of influencing brain physiology (Antal et al., 2017). So far, tDCS and tACS have been employed to enhance performance in cognitive and behavioral tasks (Fröhlich et al., 2015; Reinhart et al., 2017), as well as treat neuropsychiatric disorders such as depression, Alzheimer's, Parkinson's, stroke, schizophrenia, and many more in clinical trials (Lefaucheur et al., 2017; Elyamany et al., 2021). While progress has been significant, challenges remain, including inter-subject variability, sub-optimal stimulation parameters, and lack of long-term effects (Bland and Sale, 2019; Ammann et al., 2017; Strube et al., 2016; Wiethoff et al., 2014). This Research Topic focused on tES progress and how it may shape future behavioral and cognitive applications as well as therapeutic use.

## Mechanisms of tES: current progress

The basic physiological mechanisms of tES have been established in animal (Ranieri et al., 2012; Rahman et al., 2013; Krause et al., 2019; Wischnewski et al., 2024), human (Nitsche et al., 2005; Mosayebi-Samani et al., 2023; Woods et al., 2016), and computational models (Bikson et al., 2015; Bonaiuto and Bestmann, 2015). However, the exact mechanisms by which these effects lead to behavioral modulation are still lacking. In this Research Topic, four potential mechanisms of tACS-induced after-effects were discussed by Agboada et al.: spike-timing, spike-phase coupling, homeostatic, and state-dependent plasticity. Further, the tACS study by Carrasco-Gómez et al. reported stimulation-induced plasticity that agrees with the theories discussed by Agboada et al.. Three papers - Chen et al., Muccio et al., and Wu et al., reported plasticity induced by tDCS. However, as revealed by Wu et al., the different mechanisms by which different tES techniques operate mean their combinations might not always result in the desired after-effects.

**Figure 1:**
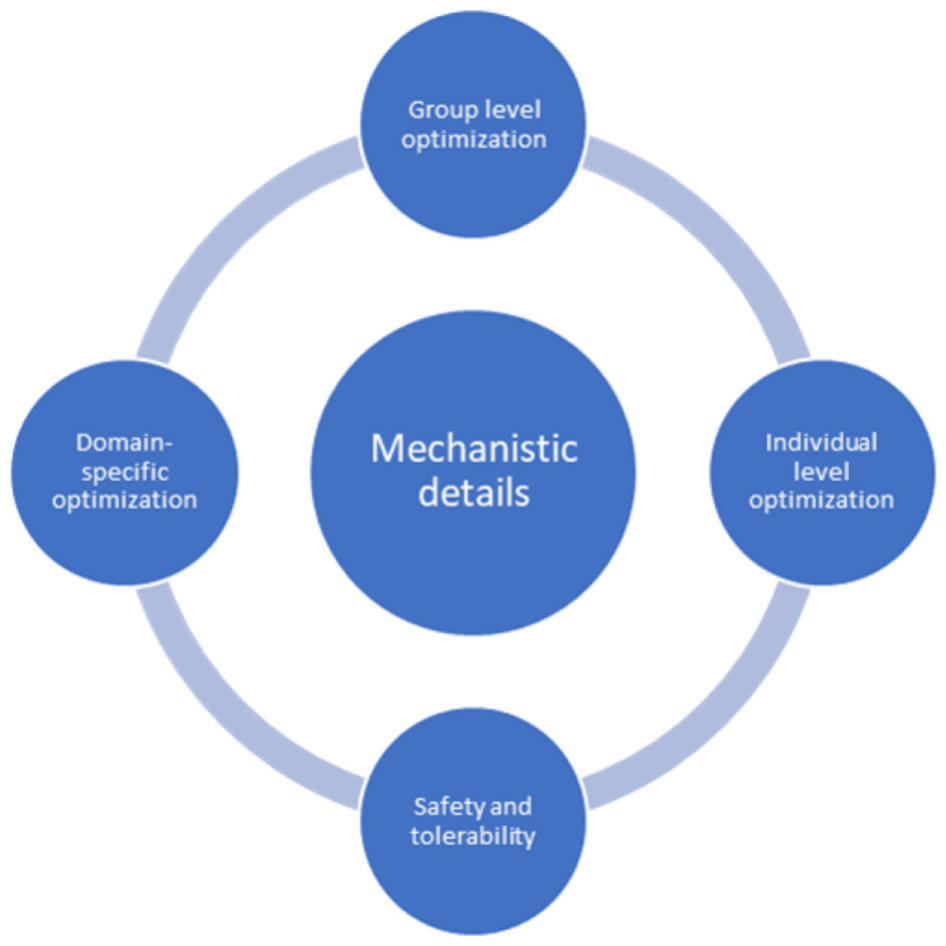
A diagrammatic representation of tES experimentation in the future.

At the center of future tES studies is the continuous investigation of the mechanistic processes underlying observed after-effects. When optimizing tES at the individual and group levels, domain-specific aims must inform safety and tolerability considerations (Figure 1).

## Clinical applications and the future of tES

In this Research Topic, Chen et al. explored the rehabilitative effects of tDCS and exergames on smartphone addiction combined with electroencephalography. TDCS improved executive control and decision-making abilities and increased P300 amplitudes in the frontal, central, and parietal cortical regions. These changes were stable over a 4-week follow-up period. Similarly, using functional neuroimaging to test the simultaneous and cumulative effects of tDCS in multiple sclerosis patients, Muccio et al. found that tDCS acutely enhanced metabolic activity, which persisted post-stimulation. At follow-up after 20 sessions of home-based tDCS with an adaptive cognitive task, the authors reported sustained after-effects of the stimulation. These studies emphasize the importance of neurophysiological evidence of tES effects, which offers mechanistic details about the stimulation efficacy. Currently, only a handful of clinical trials have measured neurophysiological and clinical measurement outcomes. Clinical studies with tES should therefore utilize a multi-modal paradigm to correlate brain and behavioral/clinical changes. Furthermore, in a pre-registered clinical trial, Xue et al. presented a protocol for assessing the effects of tDCS in patients with post-operative delirium after elective hip fracture surgery. They plan to recruit 160 patients over the age of 65 years. Using functional near-infrared spectroscopy for evaluating brain metabolic changes before and after tDCS, the authors will explore the efficacy of the stimulation in lowering post-operative delirium.

The future of tES lies in the optimization of stimulation parameters at the individual and group levels through different experimental and computational approaches (Zrenner and Ziemann, 2024). One potential individualized approach to modulate alpha oscillations applied by Carrasco-Gómez et al. used MEG to optimize tACS frequency. Also, for clinical use, tES must induce long-term after-effects (Agboada et al.). This is particularly relevant since the relatively low side-effects of tES compared to pharmacological alternatives could enforce its long-term therapeutic application (Matsumoto and Ugawa, 2017). This means reporting adverse side-effects and tES tolerability by each study to collect relevant information on how stimulation interacts with specific domains (Bikson et al., 2016). For example, Bjekić et al. compared the subjective rating of tES side-effects among healthy participants. Almost all participants (more than 95%) reported less discomfort across all tES conditions; however, when compared with sham, tACS showed slightly lower levels of discomfort than tDCS and oscillatory tDCS.

This Research Topic's collection offers a snapshot of the progress in understanding and optimizing tES in both basic and clinical neuroscience. By exploring the mechanisms of action, safety, tolerability, and clinical applications, this Research Topic highlights the potential of tES to modulate brain activity and improve outcomes in cognitive, behavioral, and neurological domains. The insights presented here, ranging from behavioral experiments in healthy human subjects to clinical studies provide a comprehensive framework for advancing scientific knowledge and translating it into practical strategies for therapeutic interventions. As we continue to refine tES protocols, personalize stimulation parameters, and investigate long-term after-effects, the research presented in this topic is essential for shaping the future of tES research and its clinical application.
